# Prenatal Diagnosis of Uniparental Disomy in Cases of Rare Autosomal Trisomies Detected Using Noninvasive Prenatal Test: A Case of Prader–Willi Syndrome

**DOI:** 10.3390/diagnostics13040580

**Published:** 2023-02-04

**Authors:** Da Kyung Hong, Ji Eun Park, Kyung Min Kang, Sung Han Shim, So Hyun Shim, You Jung Han, Hee Young Cho, Dong Hyun Cha

**Affiliations:** 1 Department of Obstetrics and Gynecology, CHA Gangnam Medical Center, CHA University, Seoul 06125, Republic of Korea; 2 Genetic Laboratory, Fertility Center of CHA Gangnam Medical Center, CHA University, Seoul 06125, Republic of Korea

**Keywords:** noninvasive prenatal test, uniparental disomy, Prader–Willi syndrome, methylation analysis, rare autosomal trisomy, amniocentesis, karyotype, imprinting disorder, trisomy rescue

## Abstract

Rare autosomal trisomies (RATs) other than common aneuploidies can be detected using noninvasive prenatal testing (NIPT). However, conventional karyotyping is insufficient for evaluating diploid fetuses with uniparental disomy (UPD) due to trisomy rescue. Using the diagnostic process for Prader–Willi syndrome (PWS), we aim to describe the need for additional prenatal diagnostic testing for confirming UPD in fetuses diagnosed with RATs via NIPT and its clinical implications. NIPT was performed using the massively parallel sequencing (MPS) method, and all pregnant women with RATs underwent amniocentesis. After confirming the normal karyotype, short tandem repeat (STR) analysis, methylation-specific PCR (MS-PCR), and methylation-specific multiplex ligation-dependent probe amplification (MS-MLPA) were performed to detect UPD. Overall, six cases were diagnosed with RATs. There was a suspicion of trisomies of chromosomes 7, 8, and 15 in two cases each. However, these cases were confirmed to have a normal karyotype using amniocentesis. In one of six cases, PWS caused by maternal UPD 15 was diagnosed using MS-PCR and MS-MLPA. We propose that in cases where RAT is detected by NIPT, UPD should be considered following trisomy rescue. Even if amniocentesis confirms a normal karyotype, UPD testing (such as MS-PCR and MS-MLPA) should be recommended for accurate assessment, as an accurate diagnosis can lead to appropriate genetic counseling and improved overall pregnancy management.

## 1. Introduction

The noninvasive prenatal test (NIPT) uses cell-free DNA in maternal blood and is currently the most sensitive and specific screening test for common aneuploidies [1]. Recently, the screening scope of NIPT has been expanded to cover chromosomal deletions or duplications of up to 7 Mb, as well as rare autosomal trisomies (RATs) and microdeletion/microduplication syndromes (MMSs) arising from chromosomal imbalances [2,3,4,5,6]. Approximately 0.47% of all NIPTs performed were positive for RATs [3].

However, despite its high sensitivity and specificity, NIPT is based on the placenta and not the fetus, and therefore, its ability to obtain fetal genetic information is limited, making NIPT unsuitable for completely replacing diagnostic testing [4,7]. If an abnormality is reported using NIPT, the possibility of false-positive or false-negative results due to fetal–placental discrepancies should be checked for, in addition to true homogeneous fetal trisomy. For this purpose, it is necessary to identify whether the result is due to true fetal mosaicism (TFM) or confined placental mosaicism (CPM). CPM refers to a chromosomal abnormality in the placenta, but not in the fetus. When nondisjunction occurs only in the cytotrophoblast and not in the inner cell mass, which becomes the fetus, mosaicism occurs only in the placenta. If no ultrasound abnormality is observed, approximately 97% of the RAT cases detected using NIPT indicate CPM [8]. Thus, although chorionic villi sampling (CVS) could also be performed, it is inappropriate owing to the risk of CPM [9].

Only cases of fetal mosaicism can survive, because cases of true fetal trisomy are nonviable. When RAT was detected using NIPT, approximately 1.5% were shown to be TFM [3]. When identifying RAT, cases of uniparental disomy (UPD) must be considered, which could lead to a chromosome imprinting disorder. Chromosomes 6, 7, 11, 14, 15, and 20 are UPD-related chromosomes associated with the now well-known imprinting disorders. Therefore, if these chromosomes are observed in the NIPT results, amniocentesis is recommended. The potential for false-positive and false-negative results still exists, and the possibility of discrepancies between NIPT and diagnostic testing further increases in the case of RAT [10]. Although amniocentesis is performed to verify fetal karyotyping, conventional karyotyping is insufficient to evaluate diploid fetuses with UPD.

**Figure 1:**
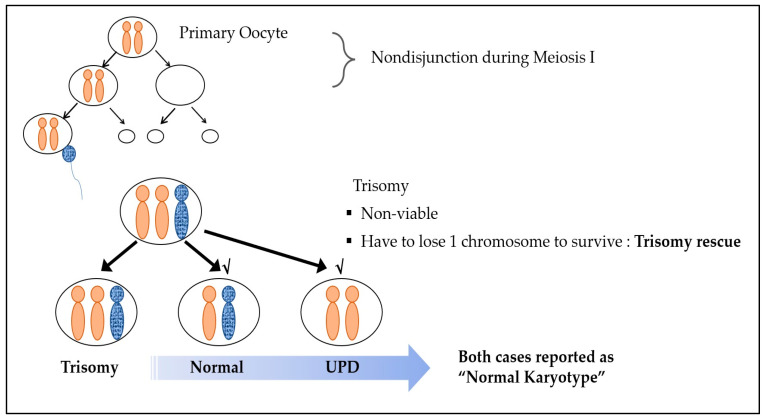
Trisomy rescue mechanism.

Nonviable trisomy conception is caused by chromosomal nondisjunction events during embryologic division. Reversion to the viable disomic state is due to “trisomy rescue”, which is the most common explanation for UPD [4,11]. In general, one pair of chromosomes should be received from each parent. However, when the disomic oocyte, which was formed via maternal nondisjunction in meiosis I, undergoes fertilization with a normal sperm cell, a trisomic conceptus is produced, and, in some cases, one chromosome is eliminated as a defense mechanism for survival. This process is called trisomy rescue. Uniparental disomy (UPD) is when the normally separated chromosome of one parent is removed, leading to both chromosomes being of the same parental origin [11] (Figure 1). Even in the case of such UPD, a normal karyotype is observed, as the chromosome number remains unaltered.

Generally, UPD is not problematic. However, UPD in some of the aforementioned chromosomes may lead to phenotypical abnormalities. Prader–Willi/Angelman syndrome, caused by an abnormality of chromosome 15, is one of the well-known abnormalities.

The proximal long arm of chromosome 15 (15q11.2-q13), localized to a 5–6 Mb genomic region, marks the Prader–Willi syndrome (PWS) region [12]. The genes in the Prader–Willi locus 15q11.2-q13 are differentially methylated and expressed only from the paternal chromosome, whereas the methylated maternally originated chromosome region is inactive. PWS is caused by the loss of function of the 15q11.2-q13 chromosomal region resulting from paternal de novo deletion, maternal chromosome 15 UPD, or the silencing of the paternal allele due to an imprinting defect (ID) [13]. Maternal UPD 15 is the second most common cause of PWS, accounting for approximately 20–30% of PWS cases [12]. The test used to diagnose PWS, such as the MS-MLPA ME028 kit, combines a quantitative analysis to determine whether there are one or two copies of the region and a methylation analysis of the region. If a deletion is suspected after this analysis, a fluorescence in situ hybridization (FISH) test is conducted to confirm the diagnosis. However, if UPD is suspected, an STR analysis will be performed [12,14,15]. This additional analysis is important for genetic counseling and verifying the genotype–phenotype connection. The American College of Medical Genetics and Genomics (ACMG) recommends prenatal UPD testing if CVS or amniocentesis results show a sample with mosaicism on one of the imprinted chromosomes, when the result is abnormal in CVS but normal in amniocentesis, or when the transfer of a mosaic embryo is observed with aneuploidy containing one of the imprinted chromosomes [9,11].

In this study, using the diagnostic process for PWS, we aim to describe additional prenatal diagnostic testing for confirming UPD in RATs detected using NIPT and its clinical implications. This includes karyotyping as the initial step in confirming the presence of CPM, followed by testing for UPD. The presented case illustrates the importance of confirmation using a UPD test in the event of abnormalities in specific chromosomes (chromosome 15, in this case), especially if amniocentesis results show a normal karyotype.

## 2. Materials and Methods

### 2.1. Study Subjects

Between July 2018 and June 2020, a total of 1138 women who underwent NIPT at CHA Gangnam Medical Center and 245 women who visited the same medical center with results indicating high-risk trisomy on NIPT from other medical centers were reviewed. Six patients with RATs were included in this series. This study was conducted after approval from the Institutional Review Board (IRB) of CHA Gangnam Medical Center, CHA University. Informed consent was waived due to the retrospective nature of the study by the IRB of the CHA Gangnam Medical Center, CHA University.

### 2.2. NIPT

NIPT was performed using the massive parallel sequencing (MPS) method and analyzed for whole-genome sequencing data. Approximately 10 mL of each maternal peripheral blood sample was collected in a Cell-Free DNA BCT™ tube (Streck, Omaha, NE, USA). Plasma was separated through centrifugation and transferred to microcentrifuge tubes. Using the QIAamp Circulating Nucleic Acid Kit (Qiagen, Hilden, Germany), plasma cfDNA was extracted from 1 mL of plasma [16]. RATs, MMS, and chromosomal deletions or duplications of up to 7 Mb as well as common trisomies (T18,13,21) and sex chromosome aneuploidies (SCAs) were identified through NIPT. For chromosomal abnormalities excluding common trisomies and SCAs, the results were provided if the patient wanted them to be reported. All pregnant women who showed RAT in their NIPT results underwent amniocentesis.

### 2.3. Methylation Analysis

**Figure 2:**
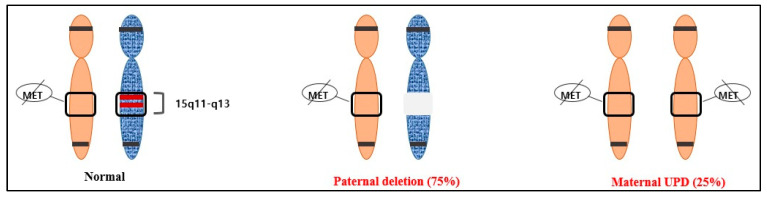
The two most common causes of PWS and their average frequencies: paternal deletion (75%) and maternal UPD (25%).

After confirming the normal karyotype using amniocentesis, methylation analysis was performed. To confirm UPD, the methylation patterns within 15q11–q13 were identified using molecular genetic methods such as methylation-specific PCR (MS-PCR) and methylation-specific multiplex ligation-dependent probe amplification (MS-MLPA). MS-MLPA is an experimental method that can provide accurate results in most cases, but it is sensitive to the type or condition of the sample, and therefore, false-negative or false-positive results may occur. Considering that, we additionally performed MS-PCR. These methods identify the methylation status of the so-called differentially methylated regions (DMRs) of the chromosome containing the gene of interest. DMRs maintain the parent-specific expression of imprinted genes and exist in different methylation states in maternal and paternal homologs [17]. MS-PCR and MS-MLPA distinguish between the normal methylation of DMRs of the chromosome of interest and abnormal loci associated with imprinting disorders, even in the absence of parental samples [11]. Currently, the most widely used assay targets the CpG island at the 5′ end of the *SNURF-SNRPN* gene, commonly called *SNRPN*, and can identify PWS in more than 99% of cases. This region is the DMR of PWS, which is not methylated in the paternally inherited expressed allele but methylated in the maternally inherited suppressed allele [12,18] (Figure 2).

MS-PCR uses the property that methylated C remains unchanged when treated with Bisulfite (Bi-S), but unmethylated C changes to T. This is a method of amplifying Bi-S-treated DNA with a maternal primer capable of amplifying the methylated *SNRPN* gene and a paternal primer capable of amplifying the unmethylated *SNRPN* gene [19]. Using MS-PCR, we analyzed the 5-CpG island of exon 1 of the *SNRPN* gene.

MS-MLPA was performed using the Probemix ME028 Prader-Willi/Angelman kit (MRC-Holland, Amsterdam, The Netherlands).

This probe mix contains 49 probes with amplification products between 129 and 481 nucleotides. There are 36 targeting probes, 8 of which contain a HhaI recognition site specific to the PWS/AS region of chromosome 15q11 and its surrounding sequences. All probes enable the detection of copy number changes in this region. Additionally, 11 reference probes that are not affected by HhaI digestion and detect genes outside the 15q11 region are included.

The MS-MLPA technique proceeds with denaturation and hybridization by mixing the sample DNA with the MLPA probe. Subsequently, the sample is divided into two tubes. Standard MLPA proceeds in one tube, resulting in the occurrence of ligation. The reactants are not cut. Through this process, copy number changes can be confirmed. In another tube, the reactants are ligated and digested with the methylation-sensitive restriction enzyme HhaI. This process gives information about the methylation ratio after digestion. Additionally, the P245 Microdeletion Probemix (MRC-Holland, Amsterdam, The Netherlands) was also used.

On chromosome 15, the maternal allele is always methylated, whereas the paternal allele is always unmethylated. MS-MLPA uses this physiologically normal state. The methylation-sensitive restriction enzyme HhaI cuts unmethylated DNA. Therefore, the digested reactants do not contain amplified ligation products. In this method, the restriction enzyme HhaI specifically cuts unmethylated DNA. In the case of a normal chromosome, the copy number would be 2. The copy number ratio is adjusted to 1 and used as the reference. In addition, since the paternal allele is digested by the HhaI enzyme, the ratio after digestion becomes 0.5, which was adjusted to 1 in this study [20]. Therefore, if the adjusted ratio after digestion with the HhaI enzyme is 2 and the copy number ratio is 0.5, it can be diagnosed as PWS caused by paternal deletion. However, if the copy number ratio is 1, PWS caused by maternal UPD can be diagnosed [21,22].

## 3. Results

Six cases were diagnosed with RATs, and trisomies of chromosomes 7, 8, and 15 were suspected in two cases each. Using amniocentesis, all cases were confirmed to have a normal karyotype. Additionally, Quantitative Fluorescent PCR, Fragment Analysis using the STR marker, MS-PCR, and MS-MLPA were performed to discriminate UPD (Table 1). In one of six cases, PWS due to maternal UPD 15 was diagnosed using MS-PCR and MS-MLPA.

**Figure 3:**
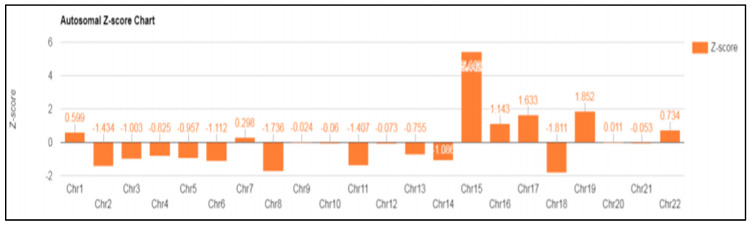
NIPT result: fetal fraction (FF): 9.659%; Z-score for chromosome 15 is 5.442.

In case 6 diagnosed with UPD, a 39-year-old patient was referred for a high risk of trisomy 15 that was observed in NIPT (Z-score: 5.442) performed around 14 weeks of gestation (Figure 3). Amniocentesis was performed around 16 weeks of gestation, and a normal karyotype was confirmed. In the case of chromosome 15, discriminating UPD was required, even though it was a normal karyotype. Therefore, the methylation of the *SNRPN* gene was analyzed using MS-PCR, MS-MLPA, and MLPA microdeletion from cultured amniotic fluid samples.

**Figure 4:**
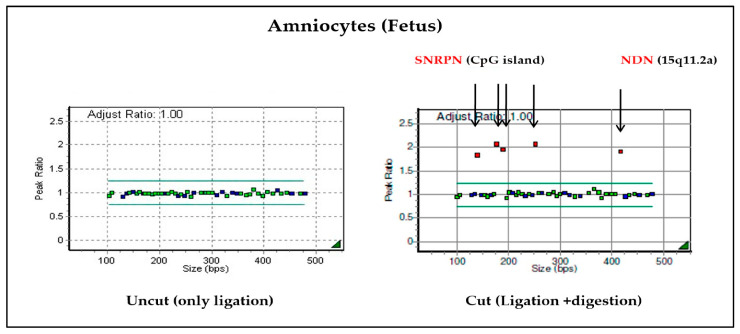
Results of MS-MLPA on chromosome 15: the *SNRPN* and *NDN* genes of chromosome 15 were not cut with enzymes, so it was confirmed that the values were doubled compared to the normal chromosome. Based on the results of these two tubes, PWS due to maternal UPD can be diagnosed.

When the cultured amniotic fluid sample was analyzed, it was confirmed that the copy number ratio was 1 in the sample from the uncut tube without the HhaI enzyme. It was also confirmed that the adjusted ratio after digestion was 2 from the tube with the Hhal enzyme, where ligation and digestion occurred (Figure 4). Therefore, it can be confirmed as PWS caused by maternal UPD. 

**Figure 5:**
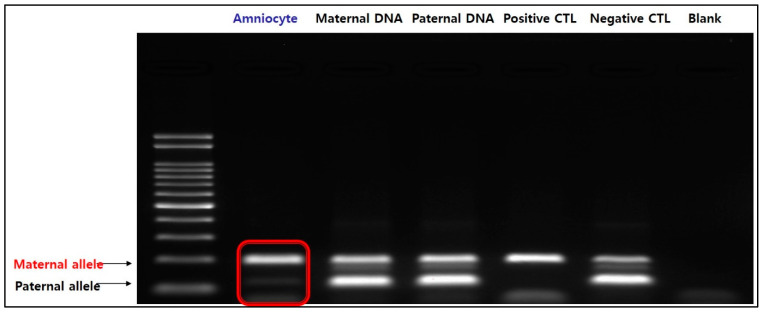
Result of MS-PCR. In amniocytes, the paternal allele is not expressed and only the maternal allele is amplified, indicating that only the maternal allele is present in the fetus.

Additionally, both maternal and paternal alleles are amplified in normal chromosome 15 (negative control) when treated with specific primers for the methylated sequence. Two distinct bands could be identified in the parental DNA; however, in the fetal amniocytes, only the maternal allele was amplified and not the paternal allele. Therefore, we confirmed that only maternal alleles are present in the fetus (Figure 5). The results of MS-MLPA and MS-PCR confirmed that this case was PWS caused by maternal UPD 15, in which the paternal allele of the *SNRPN* gene was lost.

## 4. Discussion

Rapidly developing genomic technologies are used for diagnosing the etiology of disease and malformation in the prenatal stages. In addition to common aneuploidy and sex chromosome aneuploidy, many clinical laboratories are providing genome-wide NIPT that can detect MMSs or RAT. However, a low fetal fraction or CPM confounds NIPT results, and biological factors hinder the correct interpretation of test results.

The U.S. Food and Drug Administration warns about the danger of the improper use and interpretation of NIPT results [7,23], and ACMG guidelines [24] do not recommend prenatal testing for aneuploidies other than common trisomies such as trisomies 21, 18, and 13. It also recommends caution when reporting the scope of results until improved efficacy is demonstrated in a clinical setting with large cohorts. However, high-risk NIPT findings have recently been found to be potential markers of UPD [25]; therefore, the clinical value of the test for rarer chromosomal disease syndromes remains controversial [26,27].

In the absence of other options for screening for chromosomal disease syndromes in which no visible abnormalities are detected on antenatal fetal ultrasonography [28], reports of MMSs or RATs that can be screened with NIPT may allow for additional confirmatory testing. This could affect the course of pregnancy and the prognosis, reduce the incidence of newborns with abnormalities, and reduce the family risk.

Although the RAT rate is relatively low, pregnancies identified as high risk for RAT using NIPT showed poorer outcomes, such as phenotypic abnormalities, growth restriction, preterm birth, and low birth weight, in addition to increased fetal loss and a higher rate of adverse outcomes, including neurodevelopmental delay and malformations [29,30,31,32,33,34].

In all cases where trisomy is corrected with disomy by trisomy rescue, a risk of UPD may exist. For the majority of chromosomes, UPD has no clinical consequences. However, abnormalities in chromosomes such as 6, 7, 11, 14, 15, and 20, which are associated with well-known imprinting diseases, could cause adverse effects on the offspring [3]. Therefore, an accurate diagnosis via a UPD test is required, even if amniocentesis results are normal.

PWS caused by an abnormality in chromosome 15 leads to a genetic syndrome that causes life-threatening obesity with a frequency of 1 in 10,000–20,000. PWS would increase the likelihood of the abnormal position of the fetus during delivery, assisted delivery, or cesarean section [35]. Although fetal hypomobility, polyhydramnios, and an abnormal extremity position can be observed on prenatal ultrasonography, a diagnosis based on these clinical features does not conclusively indicate PWS [36]. Making an accurate diagnosis and knowing the genetic etiology are important to discuss and prepare for the clinical course and prognosis of the condition. Counseling must also be provided to explain the genetic risk of recurrence in the affected family later on [11] (Table 2).

Chromosomal microarray (CMA) is increasingly being used as a tool for prenatal diagnosis in clinical practice and enables the genome-wide detection of chromosomal abnormalities using microarray-based comparative genomic hybridization (aCGH) or single-nucleotide polymorphism (SNP) array. These platforms allow the identification of SNP changes as well as submicroscopic deletions and duplications, called copy number variants (CNVs) [37]. It has also been reported that a correlation with UPD can be confirmed when allele homozygosity of a certain size or higher (>13.5 Mb) is shown with CMA combined with SNPs (CGH + SNP array) [38]. However, not all cases of UPD or balanced chromosome rearrangement can be detected; additionally, the interpretation of microarray results may be incorrect in the case of homozygous mutations or mosaicism [39].

DNA methylation analysis is the most effective way to confirm the genetic makeup when UPD is suspected. However, although these methods diagnose more than 99% of PWS cases, they do not discriminate between all molecular classes. In the case of MS-PCR, FISH or microsatellite analysis is required for additional confirmation, and MS-MLPA also requires microsatellite analysis to distinguish UPD from ID [12,13,40].

Excess information can increase the false-positive rate, lead to unnecessary invasive testing, and increase maternal anxiety. Currently, ACMG states that there is insufficient evidence for the routine use of NIPT to identify RATs. This may be due to the lack of clinically relevant evidence [24]. However, even with doubts about the clinical utility of NIPT, the range of chromosomal abnormalities that can be identified with NIPT is also expanding.

According to a recently published meta-analysis [41], the positive predictive value (PPV) of NIPT in RAT diagnosis was identified to be approximately 11% despite its relatively high sensitivity and specificity. Therefore, the results of the screening test could not be used as the sole basis for critical clinical decisions; additionally, the limitation of NIPT as a screening test should be explained to patients. Though the incidence rate is not high, if RAT is reported using NIPT, the chromosome associated with the imprinted gene must also be confirmed. If the amniocentesis is normal, depending on the affected chromosome, a UPD test is recommended. This will help in specific genetic counseling and overall pregnancy management.

## 5. Conclusions

In conclusion, in cases with RAT detected by NIPT, UPD following trisomy rescue should be considered. In most cases, UPD events do not result in clinical syndromes; however, chromosomes 6, 7, 11, 14, 15, and 20 have regions with parental-specific gene expression, known as imprinting. UPD of these chromosomes could lead to clinically apparent phenotypic effects. Thus, even if the amniocentesis result shows a normal karyotype, UPD testing (such as MS-PCR and MS-MLPA) is recommended in those cases for accurate assessment. An accurate diagnosis can lead to proper genetic counseling and overall pregnancy management.

## Figures and Tables

**Table 1:** Genetic testing used in six cases and results.

	NIPT Result	Karyotyping Confirmed Using Amniocentesis	Method	UPD Test Result
Case 1	Trisomy 7	Normal	MS-MLPA analysis. Probe set: ME032-A1 UPD7-UPD114/MS-PCR	No genomic imbalances
Case 2	Trisomy 7	Normal	MS-MLPA analysis. Probe set: ME032-A1 UPD7-UPD114/MS-PCR	No genomic imbalances
Case 3	Trisomy 8	Normal	STR markers: D8S264, D8S1106, D8S1104, D8S591, D8S1127, D8S1179	UPD not detected
Case 4	Trisomy 8	Normal	STR markers: D8S264, D8S1106, D8S1104, D8S591, D8S1127	UPD not detected
Case 5	Trisomy 15	Normal	MS-MLPA analysis. Probe set: P245 microdeletion/ME028 Prader-Willi/Angelman MS-PCR (Region: exon1 5-CpG island of the SNRPN gene)	No genomic imbalances
Case 6	Trisomy 15	Normal	MS-MLPA analysis. Probe set: P245 microdeletion/ME028 Prader-Willi/Angelman MS-PCR (Region: exon1 5-CpG island of the SNRPN gene)	Prader–Willi syndrome (maternal UPD)

**Table 2:** Incidence and recurrence risk according to the cause of PWS. Data from Ramsden et al. BMC Med. Genet. 2010;11:70 [13].

Genetic Mechanism	Incidence	Recurrence Risk
De novo deletion of 15q11-q13 in the paternal chromosome	75–80%	<1%
Maternal UPD	20–25%	<1%
Imprinting defects (IDs) without deletion in the IC	≈1%	<1%
Imprinting center (IC) deletion	≈10–15% of patients with an ID	Up to 50% (if father also has an IC deletion)

## Data Availability

All of the data that this paper is based on are provided or cited in this article itself.
